# Characterization of novel human endogenous retrovirus structures on chromosomes 6 and 7

**DOI:** 10.3389/fgene.2025.1498978

**Published:** 2025-01-27

**Authors:** Nicholas Pasternack, Ole Paulsen, Avindra Nath

**Affiliations:** ^1^ Section of Infections of the Nervous System, National Institute of Neurological Disorders and Stroke (NINDS), National Institutes of Health (NIH), Bethesda, MD, United States; ^2^ Department of Physiology, Development and Neuroscience, University of Cambridge, Cambridge, United Kingdom

**Keywords:** DNA sequencing, HERV-K, HML-2, long-read sequencing, structural variants, tandem repeat

## Abstract

Human endogenous retroviruses (HERV) represent nearly 8% of the human genome. Of these, HERV-K subtype HML-2 is a transposable element that plays a critical role in embryonic development and in the pathogenesis of several diseases. Quantification and characterization of these multiple HML-2 insertions in the human chromosome has been challenging due to their size, sequence homology with each other, and their repetitive nature. We examined a cohort of 222 individuals for HML-2 proviruses 6q14.1 and 7p22.1a, two loci that are capable of producing full-length viral proteins and have been previously implicated in several cancers, autoimmune disorders and neurodegenerative diseases, using long-read DNA sequencing. While the reference genome for both regions suggests these two loci are structurally dissimilar, we found that for both loci about 5% of individuals have a unique tandem repeat-like sequence (three long terminal repeat sequences sandwiching two internal, potentially protein coding sequences), while most individuals have a standard proviral structure (one internal region sandwiched by two long terminal repeats). Moreover, both proviruses can make full-length, or nearly full-length, HERV-K proteins in multiple transcription orientations. The amino acid sequences from different loci in the same transcriptional orientation share sequence homology with each other. These results demonstrate a clear, previously unreported, relationship between HML-2 loci 6q14.1 and 7p22.1a and highlight the utility of long-read sequencing to study repetitive elements. Future studies need to determine if these polymorphisms determine genetic susceptibility to diseases that are associated with them.

## Introduction

Transposable elements (TEs) make up approximately 45% of the human genome, of which ∼8% consists of human endogenous retroviruses, or HERVs (Lander et al., 2001; Snowbarger et al., 2024). Intact HERV proviruses are similar to modern exogenous retroviruses in that they consist of gag, pro, pol and env genes flanked by two long terminal repeats (LTRs). The *pol* region encodes viral proteins used in replication such as the reverse transcriptase (RT) enzyme, integrase, and ribonuclease H (Jansz and Faulkner, 2021); *gag* (group specific antigen) encodes the viral capsid, *pro* encodes the viral protease protein, and *env* encodes the viral envelope protein (Nelson et al., 2003).

It is believed that HERVs are derived from exogenous retroviruses that infected our primate ancestors and integrated into germline cells, allowing for the element to become fixed in the genome (Stoye, 2001). As HERVs have been part of our genetic makeup for millions of years, they have accumulated numerous mutations. Thus, HERV elements in our genome may not be capable of making protein, as they lack valid open reading frames (Jern and Coffin, 2008). Moreover, intact HERV viruses have not been identified in humans, although analogous ERVs in mice are known to actively cause disease (Xue et al., 2020a). HERV-K (named for the lysine, K, tRNA primer) is a member of the beta-retrovirus-like endogenous retroviruses and has 11 subtypes, termed, human endogenous MMTV-like (HML) 1 through 11 (Subramanian et al., 2011). HML-2 integrated into our genome most recently between one to five million years ago and predates the split between humans and chimpanzees. In comparison, other HERVs are much older (around 30 million years ago) (Vargiu et al., 2016). There are between 80 and 100 nearly full length HML-2 viral elements in the human genome but many are mutated and non-functional. These insertions are scattered across the human genome, but they tend to cluster in certain regions, such as on chromosomes 1, 6, 7, 8, and 12. Several sites are transcriptionally active and contain both polymorphic and viable viral genes. Although none of the insertions have a completely intact viral genome, it has the potential to replicate in humans (Subramanian et al., 2011; Wildschutte et al., 2016; Xue et al., 2020b).

HML-2 proviruses can generate virus-like particles in cancer cells (Garcia-Montojo et al., 2018). Moreover, HERV-K *pol* transcripts were found to be increased in post-mortem brain specimens from amyotrophic lateral sclerosis (ALS) patients compared to controls (Douville et al., 2011). Additionally, HERV-K expression was found to correlate with TAR DNA-binding protein 43 (TDP-43) accumulation, a known hallmark pathology of ALS (Douville et al., 2011). A study utilizing a transgenic mouse model overexpressing HERV-K/HML-2 Env exhibited an ALS like phenotype exhibiting progressive motor deficits, muscle atrophy, with loss of pyramidal neurons in the motor cortex and anterior horn cells in the spinal cord (Li, W., et al., 2015) in the motor cortex and anterior horn cells in the spinal cord (Li, W., et al., 2015).

HML-2 7p22.1 (ERVK6) is an almost complete provirus, which exists as a tandem repeat locus (i.e., three LTR elements intercalating two separate internal regions 7p22.1a and b) in about 93% of individuals and as a single locus in the other 7% of individuals (Reus et al., 2001). Proviruses 6q14.1 and 7p22.1 are known to be among the most prevalent unfixed HML-2 proviruses in humans (Wildschutte et al., 2016). Moreover, as transcriptional differences between these loci exist, structural variants at proviruses 6q14.1 and 7p22.1 and other HML-2 provirus locations may be relevant to pathophysiology of ALS (Pasternack et al., 2024), schizophrenia (Frank et al., 2005) lupus (Stearrett et al., 2021) and several cancers (Curty et al., 2020). Detecting HML-2 provirus transcripts by short-read RNA sequencing has proven challenging due to the short read length resulting in ambiguous alignment with the reference genome. However, more advanced computational tools have been developed to address this (Jin et al., 2015; Bendall et al., 2019). More recently, Oxford Nanopore Technologies (ONT) long-read sequencing has emerged as a possible way to more accurately detect and quantify TE features (Smits and Faulkner, 2023). An HML-2 provirus is about 9 kilobases (kb) in length–much longer than the 50–300 base pair (bp) read length of traditional short-read sequencing technologies. Meanwhile, ONT can achieve the longest read length of commercially available sequencing technologies with reads typically over 10 kb and up to 2.3 megabases (Mb) in length (Amarasinghe et al., 2020). The larger read size enables accurate detection of large genetic variants within the provirus itself.

We hypothesized that there would be common, large genetic variations within HML-2 provirus regions relative to the hg38 reference genome. To study this, we analyzed a dataset of 222 samples from individuals with no known pathology for structural variants (SVs), which are genetic variations larger than 50 bases in length, within HML-2 provirus regions of interest.

## Methods

### Sample preparation and sequencing

Samples used in this study were derived from the North American Brain Expression Consortium (NABEC) (dbGaP accession phs001300. v4. p1, URL: https://www.ncbi.nlm.nih.gov/projects/gap/cgi-bin/study.cgi?study_id=phs001300.v4.p1). Information regarding the samples from this dataset can be found in Supplementary Table 1. In brief, all samples were of Caucasian ancestry and about 30% of the subjects were female and 70% were male.

The details of the Oxford Nanopore Technologies (ONT) long-read DNA sequencing have been published previously (Kolmogorov et al., 2023; Billingsley et al., 2024) and uses the Napu (Nanopore Analysis Pipeline) computational pipeline to call structural variants. As part of this pipeline, Sniffles2 (Smolka et al., 2024) v.2.3 with the default parameters was used to call reference free read-based SVs, which are genetic changes relative to the hg38 reference genome greater than 50 bases in length.

### Analysis of reference genome

Locations and sequences of reference genomic DNA sequences were obtained and analyzed using the Ensembl Genome Browser and the hg38 reference genome (Cunningham et al., 2022). Conversion to amino acid sequences was performed using The European Molecular Biology Open Software Suite (EMBOSS) (Madeira et al., 2022). Transeq and was assessed for amino acid sequence similarity using FASTA (Rice et al., 2000). FASTA was performed using the UniProt Knowledgebase reference. Since there are six possible open reading frames for each DNA sequence (3 in the forward and reverse strands) that would result in different amino acid sequences, this process was repeated for each of the six possible open reading frames. Only amino acid sequences between a start (M) and stop (*) codon that were about 40 amino acids in length or longer were analyzed. To decide the functional relevance of the matching amino acid sequence, UniProt was referenced for specific locations of functional domains within the amino acid sequence (Bateman et al., 2023). The overall schematic of our approach to analyzing the sequences at the DNA and amino acid levels are presented in Supplementary Figure 1.

### Analysis of novel insertions

To make our study more targeted and clinically relevant, we analyzed SVs within HML-2 loci that were previously implicated in ALS in a different cohort (Pasternack et al., 2024). More specifically, we focused on HML-2 proviruses 6q14.1 (hg38 coordinates chr6:77716945–77726366), 7p22.1a (hg38 coordinates chr7:4576460-4591897), 19q11 (hg38 coordinates chr19:27637590- 27646453), and 11q22.1 (hg38 coordinates chr11:101695063 -101704528), transcripts of which were previously found to be upregulated in a subset of patients with ALS (Pasternack et al., 2024). We used the same coordinates for each loci in this study to ensure we were analyzing disease-relevant loci.

First, we examined the regions of the HML-2 proviruses in question using the database of genomic variants (DGV) (MacDonald et al., 2014) to determine whether there were any known insertions within these proviruses. There were no insertions within the provirus regions according to DGV “Gold Standard Variants” track, and a description of such an insertion was not found in the literature. Since we found insertions in our SV data, these novel insertion DNA sequences were subjected to a nucleotide BLAST using the blastn NCBI webpage (https://blast.ncbi.nlm.nih.gov/Blast.cgi?PROGRAM=blastn&PAGE_TYPE=BlastSearch&LINK_LOC=blasthome). The default BLAST parameters and default *homo sapiens* reference sequence database were used, but low complexity regions were not filtered (McGinnis and Madden, 2004). Next, these sequences were analyzed for HERV-K and other repetitive element sequences using RepeatMasker (Smit et al., 2013). RepeatMasker determines whether a given sequence resembles internal (INT) protein-coding sequences, LTR sequences, or other types of repetitive elements. The amino acid-level analysis was performed as described in the “Analysis of reference genome” section. In cases where scale illustrations of SVs or provirus sequences are present, the corresponding length in bases was converted to inches, so the relative scale is maintained within each figure.

### Sequence similarity and phylogeny

Both DNA and amino acid sequences were aligned with the default parameters for DNA and protein sequences using EMBOSS MAFFT (Multiple Alignment using Fast Fourier Transform) (Katoh et al., 2002) accessed using the EBI Job Dispatcher (https://www.ebi.ac.uk/jdispatcher/msa/mafft). After alignment, phylogenetic inference via maximum likelihood utilizing IQ-TREE2 (Trifinopoulos et al., 2016; Minh et al., 2020) accessed via the IQ-TREE web server (http://iqtree.cibiv.univie.ac.at/) using the default parameters. HERV-W and HML-2 provirus 11q22.1 were used as outgroups for the DNA sequence phylogenetic tree to help root the tree. Those outgroups were not used to root the amino acid tree, as we were focused on studying the alignment of the three ORF’s for the INS’s relative to the original proviruses. IQ-TREE best fit model for protein sequence was “PMB + F + I + G4” and for DNA sequence was “HKY + F + G4.” Maximum likelihood trees were used for visualizations.

### Visualizations

R version 4.3.0 was used to generate ideograms using the RIdeogram (v 0.2.2) library in R (Hao et al., 2020; Hao et al., 2020). R version 4.1.1 was used for generating 3D pie charts were using the Plotrix (v 3.8-4) library and tidyverse (v 2.0.0) was used for calculating descriptive statistics and handling the data. Figtree v 1.4.4 was used to visualize and generate phylogenetic trees.

## Results

### Four novel insertions are present within HML2 proviruses 6q14.1 and 7p22.1

Four novel insertion (INS) sequences with the potential to encode for HERV-K proteins were discovered within reference genome regions corresponding to HML-2 loci 6q14.1 and 7p22.1a (Supplementary Table 2). Based on DNA sequence similarity, each of the four INS sequences were a significant match to the corresponding HML2 provirus; the three Ch6 INS matched 6q14.1 and the Ch7 INS matched 7p22.1a. Additionally, each of the four INS were approximately the size of a complete HML-2 provirus (around 8,300 bases or 8.3 kb’s). The HML-2 provirus regions we studied generally occurred in regions of low gene density (Supplementary Figure 2). 7p22.1 is located near the telomere of Ch7. INS were not detected within loci 19q11 and 11q22.1 in this cohort.

The reference proviral sequences could make a full-length HERV-K protein (Gag in the case of 6q14.1 and Env in the case of 7p22.1) in one reading frame in the reverse orientation (Table 1). Interestingly, 7p22.1a could make truncated Gag and Pol proteins in the other two ORF orientations while 6q14.1 could make truncated Pol and Pro in one ORF. All four insertions shared a similar property – the ability to make truncated HERV-K proteins in two or three of the three possible ORFs on the reverse strand. None of the insertions could make a full-length HERV-K protein; however, two of the Ch6 insertions could make a full signal peptide (SP) subunit of the Env protein.

**TABLE 1:** Protein coding potential for 7p22.1a, 6q14.1, and the novel insertions (INS).

Sequence ID	ORF_1	ORF_2	ORF_3
6q14.1	N/A	Pol (38%), Pro (59%)	Gag (100%), Pol (32%)
chr6_77717208_INS	Env (11%), Gag (11%), Gag (10%)	Env (18%–54% TM), RT (92%), Gag (9%)	Gag (11%), Env (8%), Env (18%–100% SP)
chr6_77720617_INS	Pol (8%), Env (11%), Pol (7%)	Env (20%–45% TM), Gag (17%)	N/A
chr6_77726299_INS	Pol (8%), Env (16%)	Gag (24%), Pol (10%), Gag (22%)	Env (16%–100% SP), Gag (8%)
7p22.1a	Env (100%)	Gag (27%), Gag (65%)	Pol (91%–85% RT 100% Rnase H 100% integrase)
chr7_4586167_INS	N/A	Env (7%), Pol (9%)	Pol (5%)

Percentages are reported as amino acid match length/total amino acid length of reference sequence in UniProt. Instances where an amino acid match corresponded to regions with clear functional significance are indicated by a dash. Abbreviations: Env, envelope; SP, signal peptide; TM, transmembrane; Pol, Polymerase, Gag, group specific antigen; and RT, reverse transcriptase.

### Most individuals differ from the reference genome sequence of the 6q14.1 and 7p22.1a proviruses

**FIGURE 1:**
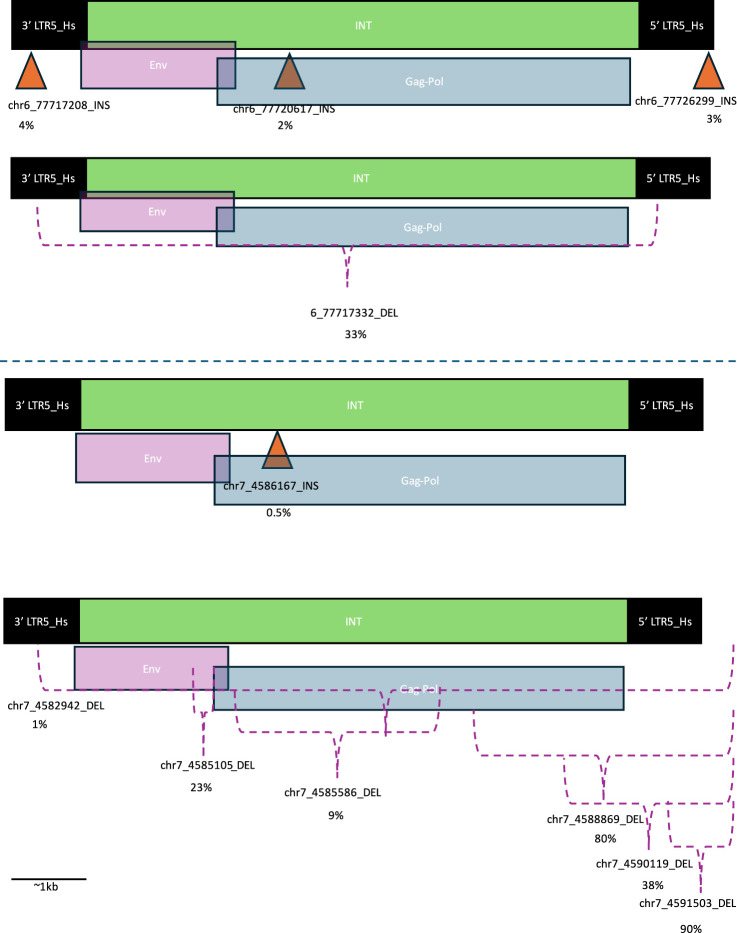
Location of structural variants within HML-2 proviruses. Orange triangles indicate location of insertions (INSs) and purple brackets indicate area of deletions (DELs). The DELs that extend beyond the original provirus are indicated by a bracket extending beyond the 5′ LTR. The percentage below each structural variant identifier (SV ID) is the percent of individuals with that SV. Long terminal repeats (LTRs) are indicated with black boxes, HERV-K internal (INT) regions are indicated with green bars and are overlayed with envelope (Env) coding regions in purple and group specific antigen-polymerase (Gag-Pol) coding regions in blue. These designations were determined based on the GenBank viewer from within the BLAST results webpage. The top proviral structures of each pair correspond to INSs, while the bottom correspond to DELs. The top two structures correspond to Ch6, while the bottom two structures correspond to Ch7.

To investigate the functional relavance of the SVs we detected within HML2 proviruses of interest we determined the location of the SVs within the functionally annotated hg38 provirus (Figure 1).

A deletion (DEL) spanning the entirety of the protein coding regions of 6q14.1 was present in about one-third of the individuals in this cohort. Meanwhile, rates of INSs were much less common – about 3%. In this cohort, the most common genomic location for INSs (4% of samples in cohort) was in the 3′ LTR of the provirus. There were also INSs in the Gag-Pol area of the INT region and the 5′ LTR, although these were slightly less common (2% and 3% respectively) (Figure 1).

Meanwhile, almost all individuals had at least one DEL corresponding to the protein coding region of 7p22.1a (80%), while 90% had a DEL within the provirus. Additionally, less than 1% had an insertion within the protein coding region. Most DELs, including the most common 7p22.1a DEL, correspond to the 5′ end of the provirus in the LTR (Figure 1).

### Novel insertion sequences lack complete LTR and intact INT regions

**FIGURE 2:**
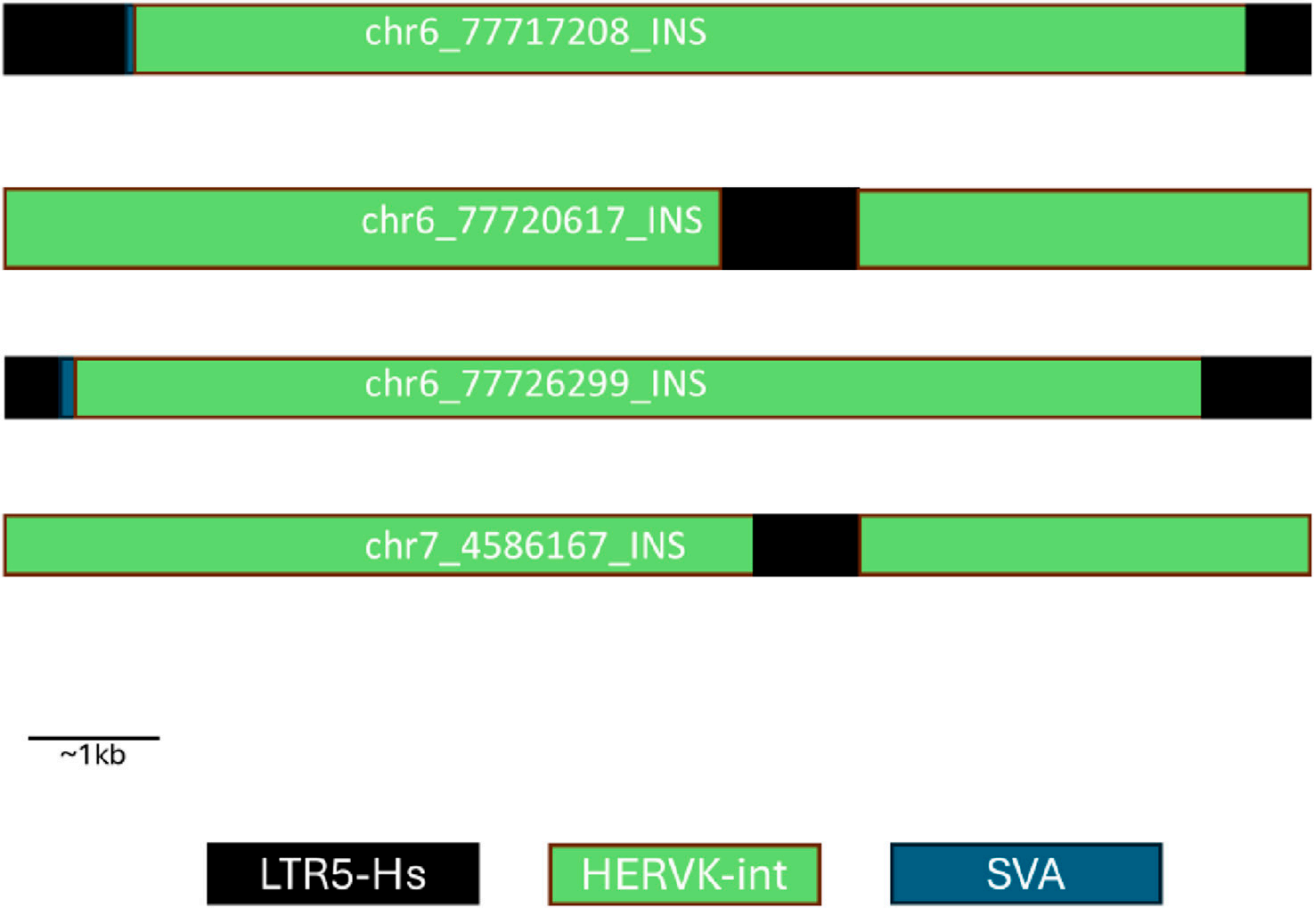
Structure of novel insertion sequences. The structure of the novel insertions, labelled by SV ID are shown. These structures were determined by running RepeatMasker on the insertion DNA sequence. SINE VNTR Alu (SVA) sequences are shown in blue, long terminal repeats (LTR) are shown in black, and HERV-K internal (int) sequences are shown in green.

The Ch7 INS lacked a typical provirus structure: instead of an INT region sandwiched by two LTR regions, it contained an LTR region sandwiched by INT regions (Figure 2). This mirrored the structure of the Ch6 INS within the 6q14.1 proviral INT region. The Ch6 INS that corresponded to the proviral LTR regions all had more typical structures: an INT region sandwiched by LTRs. Both INSs also had some sequences corresponding to SVA near the LTR, but given the short length of these, they may have been misclassified by RepeatMasker. Additionally, the length of the LTRs were variable and many were truncated.

### DNA and amino acid sequences of novel insertions have differing patterns of sequence similarity

**FIGURE 3:**
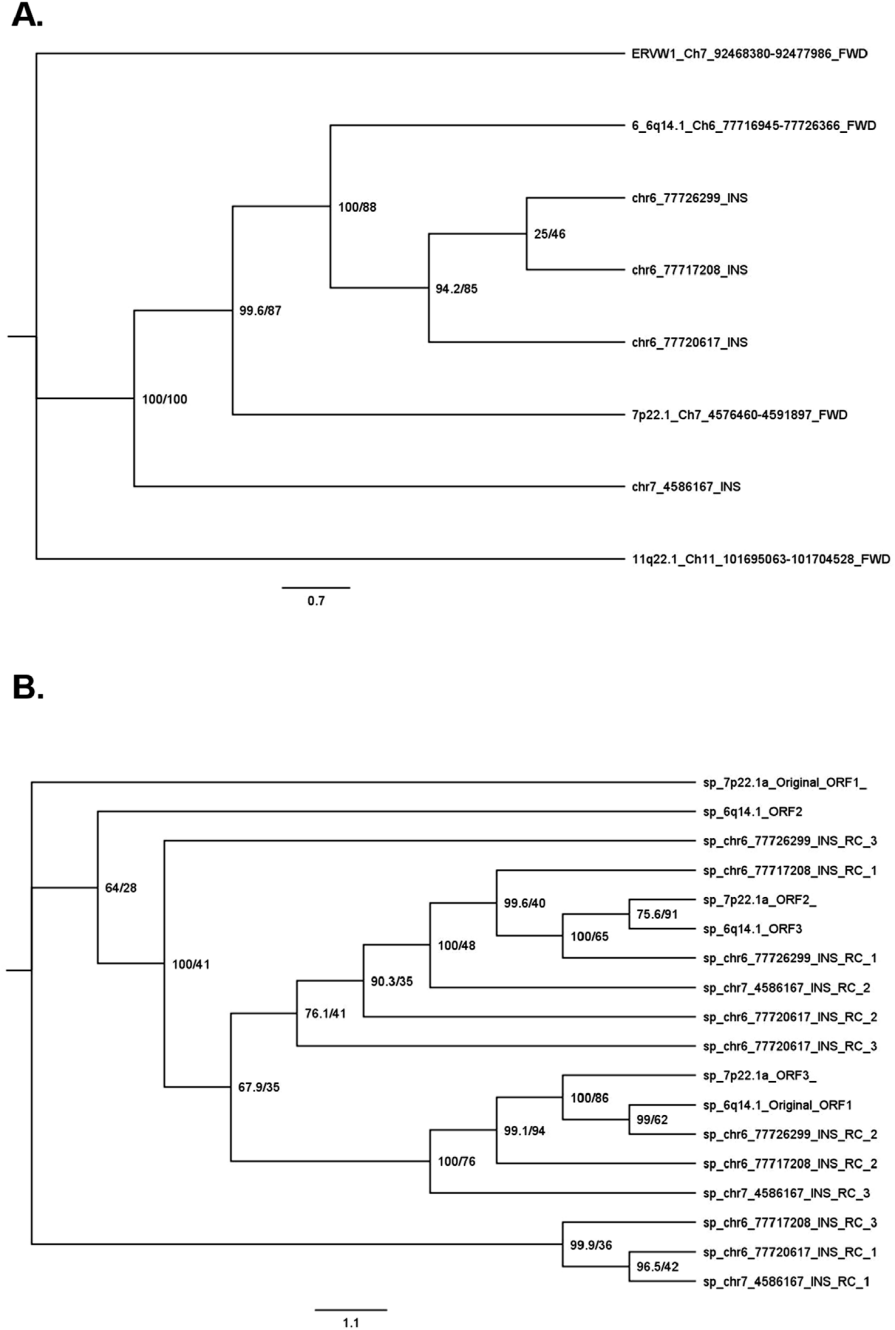
Phylogenic tree of novel insertion sequences. Sequence similarity was determined by performing an alignment with EMBOSS Multiple Alignment using Fast Fourier Transform (MAFFT) followed by IQ-TREE2 phylogenetic tree generation. Phylogenies were performed for DNA sequences **(A)** and amino acid sequences **(B)**. For the DNA phylogeny, INS are named via SV ID and proviruses are labelled by hg38 chromosomal band number, chromosomal location, and forward strand (FWD). For the amino acid phylogeny, sequences are named by chromosomal band number and open reading frame (ORF) for proviruses and SV ID and ORF relative to the reverse complement (RC) sequence. All amino acid sequences that could generate an appreciable amount of HERV-K protein could only do so on the reverse strand (RC of forward strand). Support values in percentages (%) are noted next to each node for SH-aLRT/ultrafast bootstrap.

To determine the relationship between the INS sequences and the original provirus sequences, we performed a phylogenetic analysis (Figure 3A). In terms of the DNA sequences, the three Ch6 INSs were most similar to each other: the LTR INSs were the most similar, followed by the INSs in the INT sequence, and the original 6q14.1 provirus. The original 7p22.1a provirus was more similar to the Ch6 sequences than the Ch7 INS.

The pattern of the sequence similarities of the corresponding amino acids was more complex (Figure 3B). In general, as expected, amino acid sequences from the same locus, but in different ORF orientations, were not similar to each other. For example, ORF1 of 7p22.1 is on the other side of the phylogenic tree compared to ORF3 of the same locus. On the other hand, sequences in specific orientations from different loci were similar to each other. For example, there was similarity between ORF1 of the Ch6 and Ch7 INSs with the LTR region in the middle (Figure 3B bottom); ORF2 of the same Ch6 and Ch7 INSs (Figure 3B middle), and ORF1 of the two Ch6 INSs with the INT region in the middle (Figure 3B top).

## Conclusion

In this cohort, about 95% of samples had at least one, and 80% of samples had at least two, DELs over 290 bases in length within 7p22.1a. Additionally, about 36% of samples had a DEL or INS over 8.4 kb in length within 6q14.1. At least in this cohort, variation from the reference genome seems to be the rule rather than the exception. While this may initially seem surprising, it is largely in line with previous findings: according to DGV, there are 21 structural variants within 7p22.1a including 8 deletions, three duplications, and 10 copy number variations (CNVs). The DELs range in frequency from ∼1–75%. For 6q14.1, there were 15 structural variants according to DGV including 6 deletions, one 2 bp insertion, one duplication, and 7 CNVs. The DELs range in frequency from ∼0.01–100%. Many of the DELs we identified match ones in DGV. For example, chr7_4588869_DEL from our study matches nsv508442 from DGV: both are DELs corresponding to a similar region within the provirus and both affect ∼75% of samples. We found new INSs as well as fewer, more common DELs compared to the SV data in DGV. This is not surprising given possible cohort-specific differences and that long read sequencing has advantages over short read sequencing at resolving low complexity regions of the genome (Li and Freudenberg, 2014).

Our study may have important implications for the understanding of human development, physiology, health and disease. Since HERVs have been implicated in a wide variety of cancers, autoimmune diseases, psychiatric and neurodegenerative diseases, careful characterization of these genetic variabilities and their expression profiles need to be assessed to fully understand the pathophysiological processes that mediate these diseases. Since the hg38 reference genome does not adequately capture the variation within HML-2 provirus regions, techniques that rely on mapping experimentally derived sequencing reads with a reference sequence, may underestimate the number of HML-2 transcripts present in human-derived samples. The functional relevance of these non-reference HML-2 provirus sequences should be investigated in future experiments. Additionally, future experiments aiming to accurately quantify HML-2 expression should do so by taking into account the genetic variation among individuals. For example, performing paired long-read DNA and RNA sequencing and using the individual’s genome as a reference by which to calculate RNA transcript abundances.

Finally, our study supports the hypothesis that 6q14.1 and 7p22.1 are both tandem repeat loci. A theoretical structure of the original proviral integration and possible recombinational deletion events (Belshaw et al., 2007) that could result in the SVs observed for these loci are shown in Supplementary Figure 3. The structural relationship between loci 6q14.1 and 7p22.1a means that they may have been derived from the same exogenous retrovirus. The evidence for this is fivefold:

1. The most common SVs in 7p22.1a are large DELs that span part of the protein coding region of 7p22.1a and part of the adjoining 7p22.1b provirus. DELs that span both 7p22.1a and 7p22.1b are rare (∼1%). DELs that span the 6q14.1 locus without a concomitant INS are more common but still relatively rare (∼32%).
2. About 80% of individuals have an 8,501-base deletion in the middle of the tandem repeat locus. In other words, they lack ∼2 kb of HERVK-int corresponding to the 5′ end of the Gag-Pol region of 7p22.1a, an LTR, and ∼5.4 kb’s of the 3′ end of the 7p22.1b HERVK-int region corresponding to Env and Gag-Pol. Therefore, about 80% of people have the equivalent of one canonical provirus (i.e., two LTRs sandwiching an Env and a Gag-Pol INT region) as opposed to the tandem provirus in the reference genome. This mirrors the 64% of samples that have the reference (single provirus) variant of 6q14.1.
3. Likewise, about 4% of individuals have a ∼8.4 kb INS within 6q14.1, forming a tandem repeat-like locus (i.e. 3 LTRs with two separate HERV-K INT regions between them). This mirrors the 5% of individuals that have the reference tandem repeat locus in 7p22.1.
4. Other HML-2 proviruses (e.g., 19q11 and 11q22.1) only had one SV within the locus (a DEL of 322 bases and 8,497 bases in 19q11 and 11q22.1 respectively). This indicates that large tandem-repeat-like proviral sequences are not present in all HML-2 proviruses.
5. Unlike typical HML-2 proviruses or canonical genes, both original proviruses, and some of the intra-proviral insertions, can make full-length or almost full-length HERV-K protein in at least two of three possible ORFs in the reverse orientation.

Limitations of our study include not having a functional correlate of the observed SVs. Our dataset is skewed towards male Caucasians. HERV genomics varies greatly among individuals from different ancestries and sexes, so the results of this study may not be generalizable to different groups of people. Future studies should aim to include paired long-read DNA and RNA sequencing data as well as data from subjects of diverse populations.

Here we characterized novel HML-2 proviral structures based on long-read sequencing data. Our results highlight the complexity of HERV genomics and the importance of utilizing an individualized approach to analysing this type of data. In addition, we highlight a previously unknown structural similarity between proviruses 6q14.1 and 7p22.1. This relationship is relevant not only to their physiological role but also to neurodegenerative diseases, several cancers and some autoimmune diseases because it highlights the importance of understanding the role of tandem repeat HML-2 loci expression and the polymorphisms at these loci may determine genetic susceptibility to these diseases.

## Data Availability

Publicly available datasets were analyzed in this study. This data can be found here: https://anvil.terra.bio/#workspaces/anvil-datastorage/ANVIL_NIA_CARD_LR_WGS_NABEC_GRU.
