# Supplementary material for: Characterization of novel human endogenous retrovirus structures on chromosomes 6 and 7
Source: Front Genet. 2025 Jan 27;16:1498978. doi: 10.3389/fgene.2025.1498978 (PMC11807958; doi:10.3389/fgene.2025.1498978)
Supplement: Supplementary file 1 [file DataSheet1.pdf]

Supplementary Results

	N	Ethnicity (%)	Percent Female	Percent Male	Age (Mean ± SD)	PMI (Mean ± SD)	HML-2 Provirus Insertions (Mean ± SD)
Samples with an INS	19	Caucasian (100)	29	71	50±25	14±10	3±1
All Samples	222	Caucasian (100)	36	64	50±27	12±8	<1

Supplementary Table 1. Comparison of meta data characteristics of samples with an INS in 6q14.1 and/or 7p22.1a with all samples.

SV ID	INS Size	N	BLAST Seq Match	BLAST Seq Identity (%)	BLAST E
chr6_77717208_INS	8.4kb's	9	6q14.1	96.6	~0
chr6_77720617_INS	8.4kb's	5	6q14.1	93.8	~0
chr6_77726299_INS	8.4kb's	8	6q14.1	97	~0
chr7_4586167_INS	8.3kb's	2	7p22.1	87.9	~0

Supplementary Table 2. BLAST results for novel insertion sequences. SV ID is in the format: "[chromosome #]_[chromosome position]_[SV Type]" based on the hg38 reference genome. N refers to the number of individuals with the corresponding insertion (INS). The most significant HML-2 provirus match with BLAST database is listed in the "BLAST Seq Match)" column along with the relevant BLAST statistics.

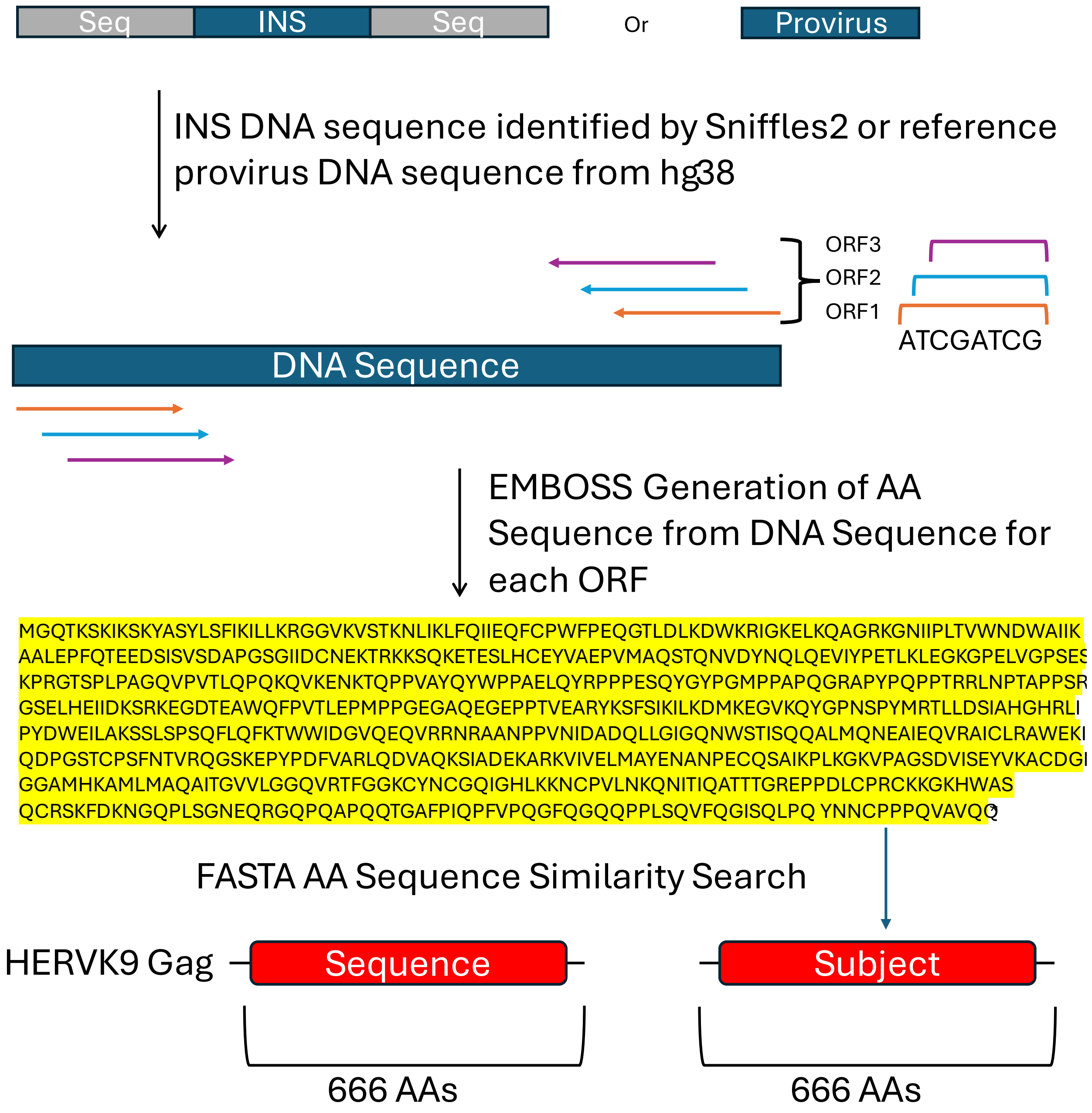

Supplementary Figure 1. Overview of analysis approach. DNA sequences were identified using Sniffles2 for novel insertions (INSs) or the hg38 reference genome sequence for canonical proviruses. These DNA sequences were then analyzed across all six open reading frames (ORFs 1-3 on forward and reverse strands). Amino acid (AA) sequences were determined based on DNA sequence using EMBOSS Transeq. These AA sequences were then analyzed via the FASTA program for significant matches to a database of known AA sequences. Representative results from 6q14.1 ORF3 were used as a basis for this figure.

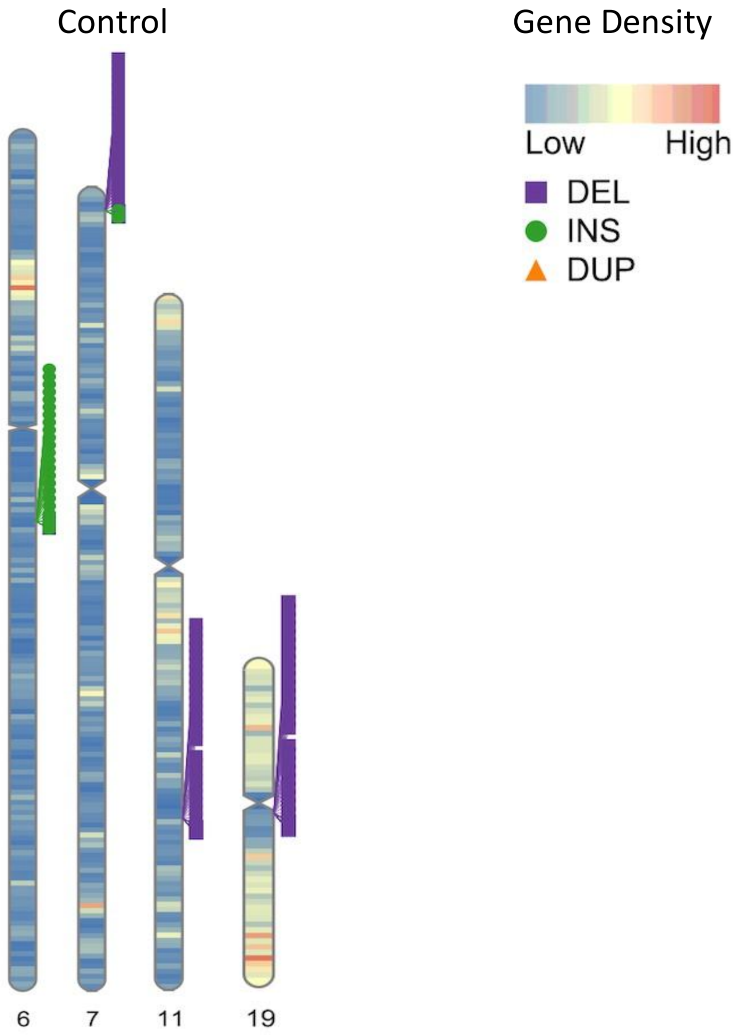

Supplementary Figure 2. Ideograms showing location of structural variants in HML-2 provirus regions along with gene densities. Ideograms were generated using the RIdeogram library in R. Color and shape of points correspond to structural variant (SV) type: deletion (DEL), insertion (INS), and duplication (DUP). The chromosomes are color-coded according to hg38 gene densities from low density (blue) to high density (red). The SVs on Ch6 and Ch7 include INS and DEL, while those on Ch11 and Ch19 are DEL only. The HML2 provirus regions studied generally occur in regions of relatively low gene density.

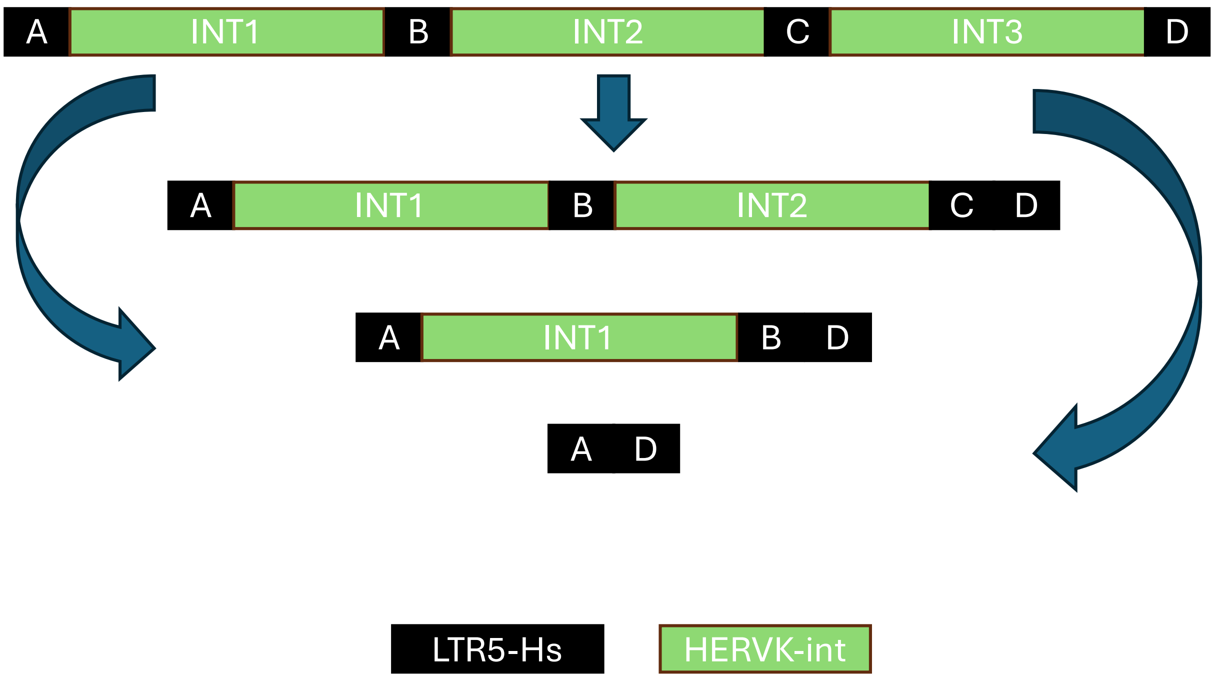

Supplementary Figure 3. Possible structure of original 6q14.1 and 7p22.1 HML2 proviruses. Arrows indicate recombinational deletion events resulting in deletions of various parts of the original provirus. Long terminal repeats (LTRs) are indicated by black boxes and HERV-K internal (INT) regions are indicated by green boxes.
